# Ferritinophagy: A novel insight into the double‐edged sword in ferritinophagy–ferroptosis axis and human diseases

**DOI:** 10.1111/cpr.13621

**Published:** 2024-02-23

**Authors:** Jing‐Yan Li, Yan‐Hua Feng, Yu‐Xuan Li, Peng‐Yi He, Qi‐Yuan Zhou, Ying‐Ping Tian, Ren‐Qi Yao, Yong‐Ming Yao

**Affiliations:** ^1^ Department of Emergency The Second Hospital of Hebei Medical University Shijiazhuang China; ^2^ Department of Orthopedics Hebei Provincial Chidren's Hospital Shijiazhuang China; ^3^ Translational Medicine Research Center Medical Innovation Research Division and Fourth Medical Center of the Chinese PLA General Hospital Beijing China

## Abstract

Nuclear receptor coactive 4 (NCOA4), which functions as a selective cargo receptor, is a critical regulator of the particularly autophagic degradation of ferritin, a process known as ferritinophagy. Mechanistically, NCOA4‐mediated ferritinophagy performs an increasingly vital role in the maintenance of intracellular iron homeostasis by promoting ferritin transport and iron release as needed. Ferritinophagy is not only involved in iron‐dependent responses but also in the pathogenesis and progression of various human diseases, including metabolism‐related, neurodegenerative, cardiovascular and infectious diseases. Therefore, ferritinophagy is of great importance in maintaining cell viability and function and represents a potential therapeutic target. Recent studies indicated that ferritinophagy regulates the signalling pathway associated with ferroptosis, a newly discovered type of cell death characterised by iron‐dependent lipid peroxidation. Although accumulating evidence clearly demonstrates the importance of the interplay between dysfunction in iron metabolism and ferroptosis, a deeper understanding of the double‐edged sword effect of ferritinophagy in ferroptosis has remained elusive. Details of the mechanisms underlying the ferritinophagy–ferroptosis axis in regulating relevant human diseases remain to be elucidated. In this review, we discuss the latest research findings regarding the mechanisms that regulate the biological function of NCOA4‐mediated ferritinophagy and its contribution to the pathophysiology of ferroptosis. The important role of the ferritinophagy–ferroptosis axis in human diseases will be discussed in detail, highlighting the great potential of targeting ferritinophagy in the treatment of diseases.

## Graphical abstract

Nuclear receptor coactive 4 (NCOA4), served as a selective cargo receptor, is critically responsible for the mediation of autophagic degradation of ferritin that is defined as ferrinnophagy. Mechanically, NCOA4‐mediated ferritinophagy is considered as a pre‐requisite for maintenance of intracellular iron homeostasis by promoting ferritin transport and iron release in accordance with requirements. Therefore, ferritinophagy is of great importance in safeguarding the viability and functionality of cells, and might be a potential therapeutic target for various diseases. In this review, we conclude the latest research findings and regulated mechanisms covering the biological function of NCOA4‐mediated ferritinophagy and its contribution to the pathophysiology of ferroptosis. Moreover, the pivotal role of ferritinophagy–ferroptosis axis in human diseases will be discussed in details, proposing to attract attention on the great potential of ferritinophagy in the treatment of various diseases.

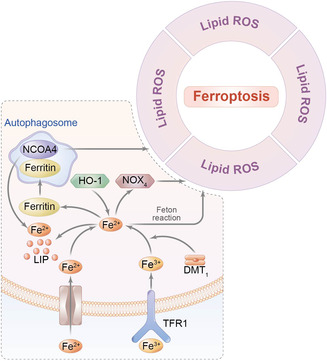

## 1 INTRODUCTION

Up to date, cellular iron, which is an essential mineral required for vital physiological responses, reportedly can influence multiple biological processes through oxygen transport, energy synthesis and DNA repair,^1^, ^2^, ^3^ which shows great benefit in ameliorating organ dysfunction and improving metabolic balance.^4^ Iron deficiency, attributed to either insufficiency of intake or dysfunction of ferritin transport, is deemed to be the major cause of structural derangement as well as aberrant function. Likewise, both structural integrity and organic stability can be impaired under exposure to iron deficiency, thereby contributing to fatal consequences.^5^ By contrast, iron overload is inclined to jeopardise organelle and even tissues owing to the production of reactive oxygen species (ROS) generated via the Fenton reaction of the Haber–Weiss cycle, a process newly defined as ferroptosis.^6^, ^7^ Therefore, the dynamic balance between iron availability and intake, together with tightly regulated crosstalk between ferritin transport, may act as an indispensable self‐protective mechanism.^8^ Ferritinophagy is a newly discovered form of autophagy related to ferritin degradation that plays a crucial role in regulating iron homeostasis with the help of nuclear receptor coactive 4 (NCOA4).^9^ In particular, this specific receptor is responsible for binding with ferritin during the initial developmental stage of the autophagic process, the consumption of which aggravates iron overload and further exacerbates disruptions in cellular function and can even survive.^10^ It is noteworthy that this process is closely dependent on intracellular iron levels and iron utilisation.^11^ Thus, disruptions in iron‐dependent ferritinophagy to varying degrees reportedly contribute to diseases affecting multiple systems, from metabolic disorders to tumour‐associated tissue damage, indicating that ferritinophagy is of great importance for cell fate. Given that NCOA4 facilitates Fe^2+^ bioavailability in the iron metabolism cycle, it is possible for the negative feedback to impact the ferritinophagy–ferroptosis axis upon excessive induction of ferritinophagy.^12^ To this end, interventions of ferritinophagy‐mediated ferroptosis might be a promising yet challenging achievement to improve prognosis in metabolic stress settings. In this review, we will attempt to summarise the biochemical role of NCOA4‐mediated ferritinophagy and the molecular machinery of the ferritinophagy–ferroptosis axis under the challenge of continuous stress or iron‐disordered vicious circle. Concurrently, potential targets for human diseases underlying the therapeutic strategy might be further concentrated to protect against ferroptotic cell death.

## 2 THE BIOLOGICAL FUNCTION OF NCOA4‐MEDIATED FERRITINOPHAGY

### 2.1 The physiological role of NCOA4

As we know, NCOA4 is identified as a selective autophagy receptor with a key role in mediating the transport of Fe‐carrier ferritin to the organelle of lysosome for further polymer degradation, followed by iron delivery, which is defined as ferritinophagy.^13^ The specific receptor was originally found to be a constituent of RET fused gene that integrally conjugated N‐terminal region to the actively structural domain of the ret oncogene named tyrosine kinase.^14^ Moreover, the gene encoding of NCOA4 protein is initially demonstrated to be located on the chromosomes of both humans (10q11.2) and mice (14B) that contain approximately 10 exons, eventually leading to a mature transcriptome of encoding amino acids.^15^ NCOA4 is composed of multilocus tautomeric forms in animals, whereas only two transcript variants in humans, involving NCOA4α (614 Aa, 70 kDa) and NCOA4β (286 Aa, 35 kDa).^11^ These isoforms, however, can bind up to the whole N‐terminal coiled‐coil domain and a fraction of C‐terminus, as evidenced by the interaction between NCOA4 gene recombination and ferritin heavy chain 1 (FTH1) projected to the NCOA4 C‐terminal domain, in which a protein transduction domain only exhibits in NCOA4α instead of NCOA4β.^16^ As a matter of fact, the deep exploration of molecular structure information facilitates the accurate understanding of the NCOA4 coiled‐coil domain, which accelerates the development in ferritinophagy research.^17^, ^18^ Intriguingly, the located domain of N‐terminal coil has been confirmed as a highly conserved region that is responsible for combining with nucleotide‐binding oligomerisation in case of autophagy triggering. In addition, more evidence suggests that the N‐terminal coiled‐coil domain of NCOA4 provides a protective function to limit genome damage in cells in response to metabolic stress.^19^

Because NCOA4 has been verified as the prime motivator in cell proliferation and differentiation, migration, invasion and even multidrug resistance to tumour cells, which relates to the progression and metabolism of cancer cells, it follows that further research in spatial conformation and variant difference of NCOA4 seems to be necessary.^20^ Interestingly, a previous report documented that NCOA4 was verified as one of the most affluent receptors in autophagosomes, manifested by increased accumulation of autophagic vacuoles and enhanced expression of quantitative mass spectrometry‐based proteins.^21^ Concomitantly, NCOA4 can potentially present with the cohesive capacity for co‐localising with the protein structural domain at the initial stage of ferritinophagy. Taking the subunit of ferritin heavy and light chains (FTH1/FTL), for example, it enables tight binding with the C‐terminal domain in NCOA4 through an immediate interplay with conservative surface arginine, which is solely presented in NCOA4α.^22^ HECT and RLD domain containing E3 ubiquitin protein ligase 2 (HERC2), another protein subunit that interacts with NCOA4 in the dynamic autophagy process, exerts a close contact with NCOA4–ferritin complex after ferritinophagy initiation, thereby contributing to functional stability.^23^ In the case of enhanced NCOA4 expression, the essential process of ferritin delivery to lysosomes will be obviously mobilised in the hyperdynamic phase, indicating a significant participation of NCOA4 in selective autophagic‐mediated ferritin degradation. In return, intracellular iron bioavailability might radically decline when the ferritin turnover rate decreases due to NCOA4 depletion, thus confirming that NCOA4 is required for regulating intracellular iron homeostasis.

**FIGURE 1:**
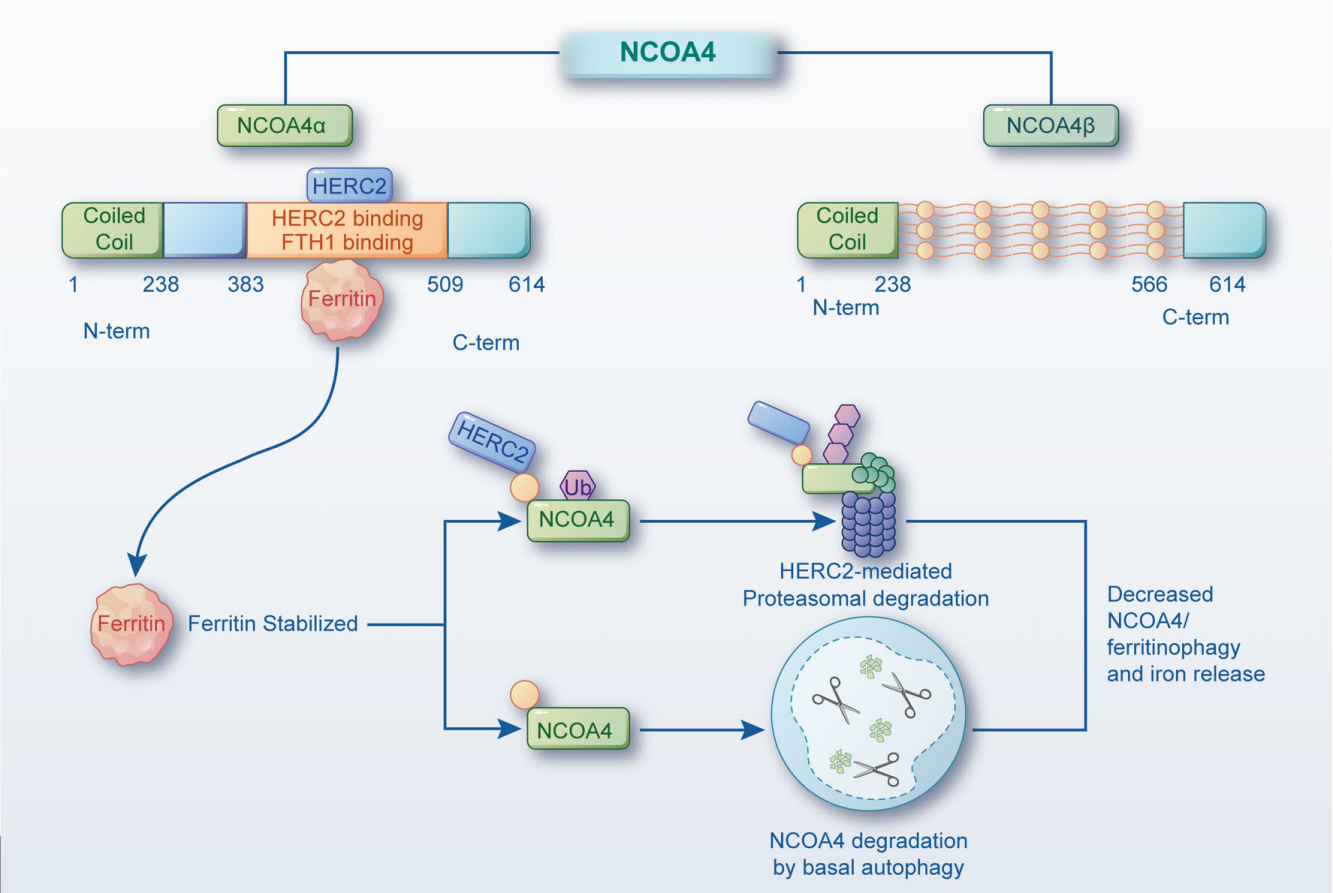
The physiological role of NCOA4. NCOA4 is composed of multiple splice variants in animals, whereas only two transcript variants in humans, involving NCOA4α (614 Aa, 70 kDa) and NCOA4β (286 Aa, 35 kDa). FTH1 combines with the C‐terminal domain in NCOA4 through a direct interaction of conserved surface arginine in NCOA4α. In consideration of NCOA4 specifically recognising FTH1 rather than FTL, the stored iron thereby converted into a mixture of FTH1–FTL that revealed a conformational transformation in the composition of ferritin complexes. The functional activation of NCOA4 is dependent on the interaction with another molecular called HERC2, which mainly targets the proteasome by virtue of structural destruction. HERC2, a large multidomain homologous to E6AP carboxy terminus (HECT) E3 ubiquitin ligase, can bind with NCOA4 for dynamic autophagy process when iron levels are high and subsequently drive close contact with NCOA4‐ferritin complex after ferritinophagy initiation to maintain functional stability. In the condition of cellular iron deficiency, HERC maintains a monosome instead of interacting with NCOA4. The high level of NCOA4 then triggers ferritinophagy, and subsequently increases cellular iron overload. Indeed, the HERC2 region spanning amino acids from 2292 to 2923 chelates NCOA4 more specifically in iron‐replete conditions than in iron‐chelated conditions. HERC2, HECT and RLD domain containing E3 ubiquitin protein ligase 2; FTH1/FTL, ferritin heavy and light chains; NCOA4, nuclear receptor coactive 4.

Further evidence has confirmed the above interaction between NCOA4 and FTH1 and subsequently verified FTH1 to share 24 NCOA4 structural fragments.^22^ Moreover, downregulation of NCOA4 can suppress FTH1 to binding with ferritin, ultimately giving rise to impairment of ferritin transport to autophagosomes. Thus, it proves that FT degradation and chelation of NCOA4 and lysosomes can be significantly inhibited under the condition of NCOA4 deficiency, which ultimately contributes to a scatter positioning pattern of ferritin.^24^ Likely, gene mutants of FTH1^R23A^ fail to transfer ferritin to lysosomes and further drive the deterioration of ferritinophagic activation under pathological conditions. In general, silencing or overexpression of NCOA4 can produce a remarkable impact on iron metabolism, which contributes to the enhanced level of FT and TFR1, eventually leading to confer resistance to peroxidative conditions. It is evidenced that NCOA4 upregulation can promote ferritinophagy while NCOA4 downregulation is regarded as an element for low bioavailability of iron.^25^ In addition, the increased co‐localisation of iron chelation and endogenous NCOA4 has been suggested for the potential relation between protein receptors and iron homeostasis. Collectively, the physiological role of NCOA4 is shown in Figure 1.

### 2.2 NCOA4 mediates ferritin transport into the lysosome for degradation

**FIGURE 2:**
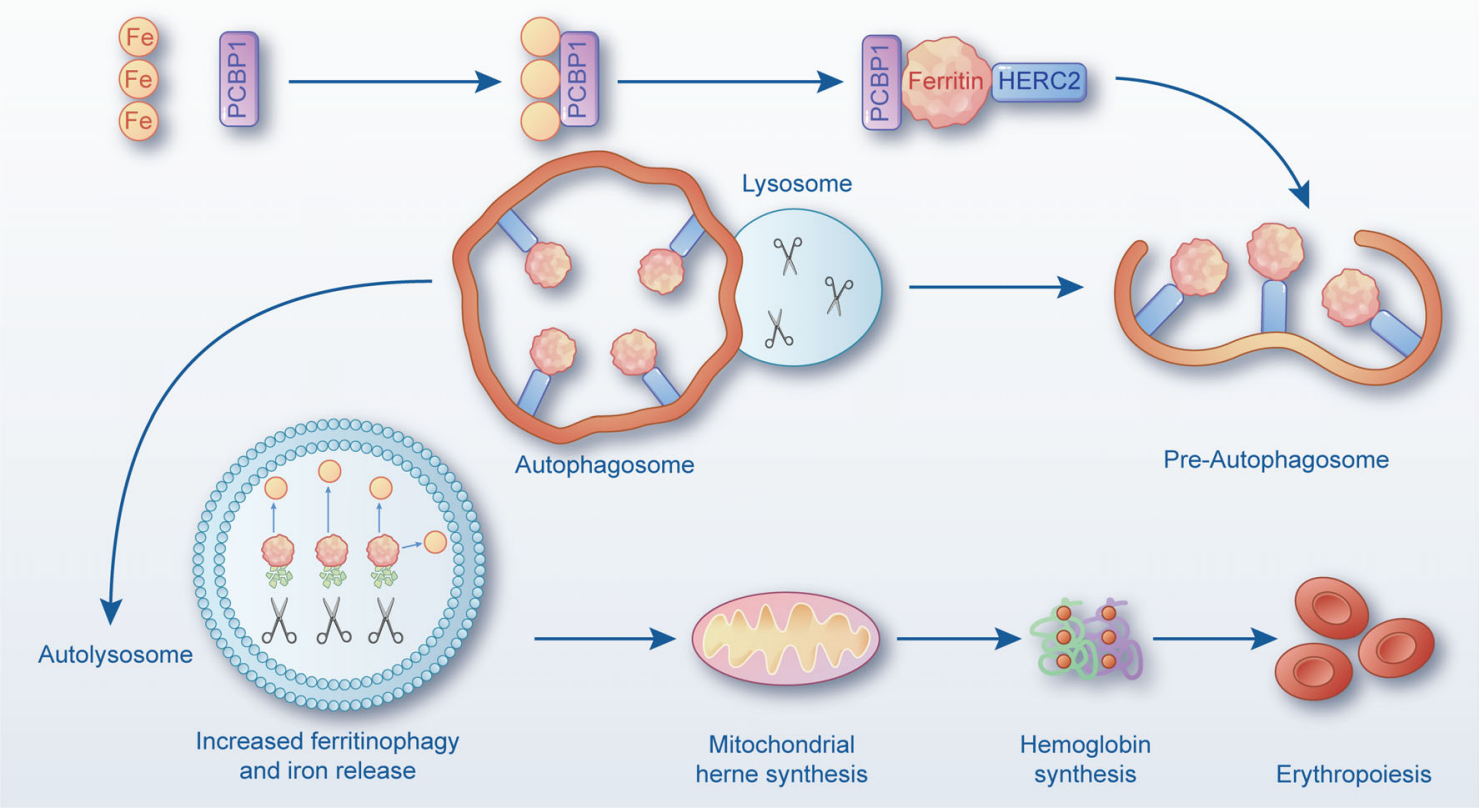
NCOA4 mediates ferritin transport into the lysosome for degradation. During iron utilisation, ferritin can be dissociated from conjoint pores with the help of a small proteasome complex. Depending on PCBP1, Fe is combined with NCOA4 and ferritin to balance iron homeostasis. Double‐membrane vesicles can be constituted in the hyperdynamic phase by coordinating with Atg proteins, which phagocytise cellular cargo, eventually leading to lysosomal degradation. Ferroportin‐regulated iron exportation facilitates ferritin degradation via the proteasome whereas iron chelation inclines to lysosomal degradation. Atg, autophagy‐related gene; NCOA4, nuclear receptor coactive 4; PCBP1, poly rC‐binding protein 1.

As the typical characteristic in the NCOA4 structural domain is deemed as the major contributor not only to the mediation of oligomerisation but also to the involvement of iron homeostasis, a growing number of attention has been distracted to the mechanisms underlying NCOA4‐mediated ferritin transport (Figure 2). As with cellular iron atoms reserved mainly in divalent metal transport, a molecular containing multiple subunits of immunoglobulin light and heavy variable region chins can be chelated via ferritin iron pores, and further oxygenised to Fe(III) by the endogenous FTH structural domain, eventually exasperating the unavailability for Fe(III) or accumulation of ROS.^26^, ^27^ During the iron utilisation process, ferritin naturally dissociates from conjoint pores with the help of minor proteasomal enzymes, which is followed by proteolytic degradation of the ferritin shell.^28^ Notably, both iron release and ferritin degradation are reportedly dependent on functional lysosome; this is due to the critical involvement of the lysosome‐related pathway in the catabolic cellular process under various lethal conditions.^29^, ^30^ Until now, various types of ferritin degradation have been reported, presenting a close contact with autophagy. This connection termed as an evolutionary conservation in catabolic process can bestow a protective effect on multiple organs and systems when faced with stress. This protective effect is achieved through the degradative ability of lysosomes, ultimately ensuring cellular homeostasis.^31^, ^32^

In general, double‐membrane vesicles are constituted in hyperdynamic phases, accompanied by coordination to a fixed pattern of autophagy‐related proteins, which phagocytize intracellular cargo in both selective and non‐selective manner, in turn, resulting in lysosomal degradation.^33^ Conversely, integration between autophagosomes and lysosomes thereby contributes to the delivery of cargo for further degradation with the help of lysosomal enzymes.^34^ It is well accepted that autophagy is a survival response to various stresses, such as nutrient starvation, hypoxia as well as ROS, and is also involved in eliminating invading pathogens for the maintenance of intracellular homeostasis.^35^ Classical autophagy is characterised by double‐membrane vesicles named autophagosomes, which are indispensable to the synergistic effect of multitudinous autophagy‐related proteins (Atg). These autophagosomes are responsible for transporting and capturing cellular content in the manner of phagocytosis, eventually leading to lysosomal degradation. In the initial phage of selective and non‐selective autophagy, both ULK activation and phosphatidylinositol 3‐phosphate generation are pre‐requisite for lysosomal degradation, which is followed by the cooperation of membrane vesicles. Lysosome‐related degradation pathways are subsequently activated by virtue of catabolic enzymes through combination of autophagosomes and lysosomes. Actually, autophagy was defined as a survival resistance against external starvation, whereas the latest assumption that autophagy is hyperactive in most cells has been proved to degrade the cellular cargo under multiple stimulations.

As advancements in the field of autophagy continue, a more precise concept known as selective autophagy (one of the major forms of autophagy) has recently shown an enhanced research upsurge. The selective autophagic processes have been verified to target specific multicellular organelles for further cargo degradation, such as endoplasmic reticulum, mitochondria, peroxisomes, as well as proteasomes or protein aggregates. It can exhibit physiological function by eliminating invaded pathogens, avoiding the overproduction of stress proteins and targeting the degradation of specific substrates, especially dysfunction or superfluous organelles.^36^, ^37^ While increasing knowledge continues to reveal that NCOA4‐mediated ferritin degradation is regarded as conservative catabolism. It has been considered to protect the intracellular environment against stress response and unstable factors by means of the degradative process of damaged organelle in the lysosome. Meanwhile, the clinical significance of ferritinophagy has been recognised since a specific receptor named NCOA4 was first identified as a cause for iron‐depleted cells by Mancias et al., indicating that selective autophagy is required for ferritin degradation via lysosome.^9^ Inherently, the intracellular iron level is responsible for tightly regulating NCOA4 expression at the initial phase, which appears to be a pre‐requisite for the ferritinophagic pathway in return.^38^ Under conditions of cellular iron abundance, for example, HERC2 (an E3 ubiquitin ligase) binds more strongly to NCOA4, thereby augmenting proteasomal degradation of NCOA4.^39^ Thus, it is inferred that the decreased NCOA4 expression induced by the inhibition of ferritinophagy might be a primary contributor to ferritin iron storage. Similar to HERC2, FTH1 is another mediator that facilitates the dissociation of the NCOA4–FTH1 complex under iron‐replete cellular conditions, eventually contributing to the suppression of ferritinophagy and ferritin iron reservation.^2^ Intriguingly, further study has demonstrated the combined action between HERC2 and FTH1 to be mutually exclusive, as evidenced by the overlapped site for HERC2 binding on NCOA4.^11^ Therefore, a decrease in the ratio of HERC2 occupancy on NCOA4 is presumably considered as a precondition to maintain NCOA4 stabilisation. Although it has been shown that NCOA4 can chelate or even co‐purify with iron, whether it binds to HERC2 and FTH1 in a synchronous or competitive manner remains to be determined.

Until now, the precise mechanism by which NCOA4 is delivered to lysosomes remains unclear, even though NCOA4 is closely related to autophagy‐related gene (Atg) 8, one of the autophagic family members that is capable of conjugating to LC3B structural domain. Considering that NCOA4 lacks a canonical LC3‐interaction region motif, researchers have speculated that alternative motifs play a role in mediating Atg binding.^40^ Aside from the classical autophagy‐associated pathway involved in NCOA4‐mediated ferritin transport, other biological pathways, such as the ubiquitin–proteasome system, play potential roles in the process of ferritin degradation. In parallel, an early report from Domenico et al. showed ferroportin‐regulated iron exportation facilitated ferritin degradation via the proteasome, whereas iron chelation inclined to lysosomal degradation, implying that degradative manner might vary from cell type in response to iron deficiency.^41^ Also, multiple alternative NCOA4‐ferritin pathways have been documented to be independent of ATG8 and lysosomal trafficking manner, including the Tax1 human T cell leukaemia virus type I, RB1‐inducible coiled‐coil 1/FIP200 and ATG9A pathways.^42^ Taking ESCRT endosomal signalling, for example, it has been well established as a highly regulated manner for ferritin degradation, which has been manifested to mediate nutritional deficiency‐related degradation of autophagic receptors.^43^ In short, future studies on an exploration of precise binding sites between NCOA4 and ferritin seem to be inevitable for further illustrating the mechanism with regard to NCOA4‐mediated ferritin transport into the lysosome for degradation.

### 2.3 NCOA4 mediates the release of ferritin iron to promote erythropoiesis

**FIGURE 3:**
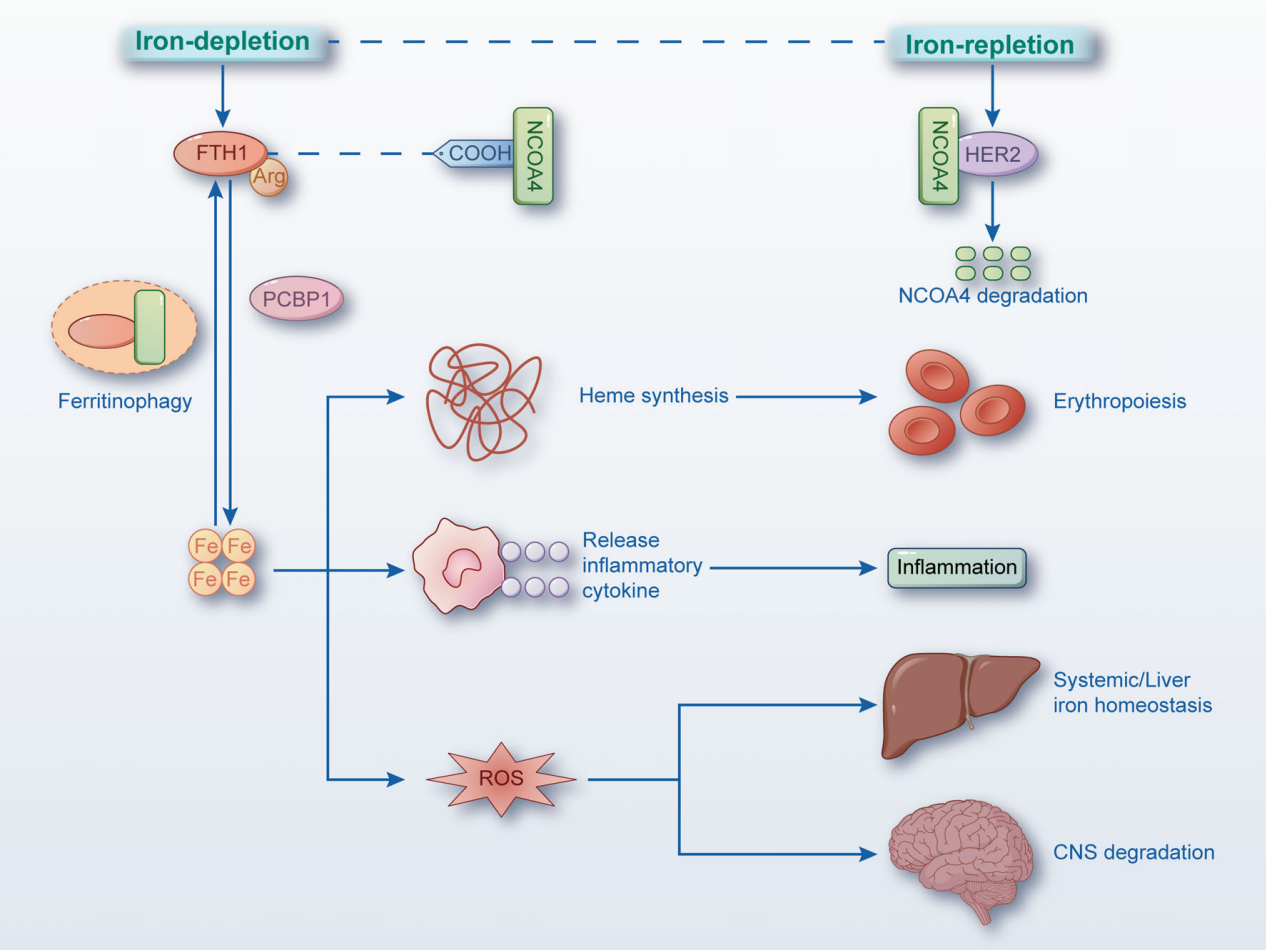
NCOA4 mediates the release of ferritin iron to promote erythropoiesis. Intracellular iron can be transported to RBC with the help of Tf and Tf‐mediated endocytosis, followed by being reduced to Fe(II) form. Along with ferritin, iron is directly delivered to mitochondrion for heme synthesis or provisionally stored in LIP, accompanied by being degraded in the lysosome and eventually driven to mitochondrion for multifunctional biology. PCBP1 and NCOA4 coordinate to supply heme synthesis with sufficient iron via ferritin and ferritinophagy at the early stage of erythroblast. During the late phage of erythrocyte, levels of iron import together with ferritins markedly decrease, and iron in endosomes might be transferred to mitochondria for heme synthesis. HERC2, HECT and RLD domain containing E3 ubiquitin protein ligase 2; LIP, labile iron pool; NCOA4, nuclear receptor coactive 4; PCBP1, poly rC‐binding protein 1; RBC, red blood cell; Tf, transferrin.

Given that the conserved function of NCOA4 appears to be an elementary yet exquisite contributor to ferritin degradation and intracellular homeostasis under an excitable response, a growing number of studies have suggested that NCOA4 is recruited to chromatin regions to further facilitate erythropoiesis in a transcriptional process (Figure 3).^44^, ^45^ As is known to all, both haematopoietic stem cell differentiation and mature erythrocytes decenucleation have been illustrated to be implicated in the pathogenesis of dysfunctional erythropoiesis, which is characterised by the gradual programmed dissociation of haemoglobin from cellular components.^46^ While most iron in the human body serves as the indispensable element for haemoglobinisation, it can be transported to red blood cells with the help of transferrin (Tf) and Tf‐mediated endocytosis followed by being reduced to Fe(II) form.^47^, ^48^ Along with ferritin, iron is directly delivered to mitochondrion for heme synthesis or provisionally stored in labile iron pool (LIP) accompanied by degradation in lysosomes and eventual transport to mitochondria for multifunctional biology.^49^, ^50^ In agreement with the above theoretical basis, a recent study investigating the key role of ferritinophagy in mitochondrial iron transport and heme synthesis revealed that deficient expression of NCOA4 is the major driver of haemoglobinisation failure in the absence of disorder in differentiation, indicating that decreased levels of NCOA4 inhibit ferritinophagy to further replenish iron stores.^51^ Another model study with respect to the temporal evaluation of haemoglobinisation reported by Philpott found that the iron chaperone named poly rC‐binding protein 1 (PCBP1) binds sequentially to NCOA4 and ferritin to balance iron homeostasis, which was up to the erythroid differentiation phase.^51^ In particular, PCBP1 and NCOA4 synergistically supply sufficient iron for heme synthesis via ferritin and ferritinophagy in the early stages of erythroblast formation. During the later phase of erythrocyte formation, however, the level of iron imported together with ferritin declines markedly, and iron in endosomes can be transferred to mitochondria for heme synthesis.

To date, the well‐studied mechanism underlying the biological function of NCOA4 in erythropoiesis has been elucidated in vivo using gene‐deficient animal models. Before the evaluation of the systemic knockout (KO) animals, transcriptional profiling of erythroblasts was utilised to determine the fundamental connection between NCOA4 mRNA expression and erythropoiesis. These studies indicated that expression of the NCOA4 is upregulated in erythroblasts for haemoglobin synthesis.^52^, ^53^ In agreement with the results of zebrafish model studies, Bellelli et al. showed that NCOA4‐mediated ferritinophagy plays critical roles in regulating iron availability in globin synthesis and haemoglobinisation, supported by hypochromic microcytic anaemia together with excessive iron in murine liver tissue, which revealed the hypomotility of iron from ferritin in the setting of *Ncoa4*‐deficient mice.^54^ In contrast, superfluous iron‐laden ferritin and accumulated cytoplasmic iron were noted in the liver when mice were conducted with the iron‐rich diet.^54^ Moreover, NCOA4‐mediated ferritinophagy was presented with a significant fluxionary temporal demand due to the more serious young NCOA4‐deficient mice compared with that of the adult mice. These data thus suggest that the failure of efficient ferritin degradation is ultimately apt to drive cells into a higher susceptibility to a peroxidative state. Indeed, downregulated expression of NCOA4 can exert an impact on ferritin transport but not on ferritin secretion, suggesting that NCOA4 is non‐essential for ferritin secretion, thereby accounting for the overloaded ferritin presented in serum of *Ncoa4*‐KO mice.^55^, ^56^ Unlike NCOA4‐mediated lysosomal delivery, both non‐classical secretory‐autophagy manner and vesica‐exosome pattern were shown to involve in the physiological process of ferritin secretion in the independent pathways until 2018.^57^

It is our belief that potential mechanisms with respect to the key role of NCOA4 in regulating erythropoiesis will be further clarified, especially for the non‐autonomous mode. As the absolute KO of NCOA4 is inevitable for cells to give rise to compensatory response and drives the haemopoietic system to confront *Ncoa4* deficiency, conditional KO of NCOA4 in bone marrow‐derived cells for in vivo experiment seems more persuasive to demonstrate the regulatory effect of NCOA4 on haemoglobin synthesis.

## 3 MOLECULAR MECHANISMS UNDERLYING NCOA4‐MEDIATED FERRITINOPHAGY IN FERRITINOPHAGY–FERROPTOSIS AXIS

### 3.1 Ferritinophagy regulates iron‐dependent peroxidation in ferroptosis

As described above with regard to quantitative proteomics analyses, NCOA4 refers to a ferritinophagic receptor that is combined with the C terminal of FTH, accounting for the specific degradation of ferritin and enhanced bioavailability of cytosol iron.^9^ There are two definite pathways in the negative regulation of NCOA4 under iron‐overloaded conditions: interaction with HERC2 to mediate ubiquitination and initiation of autophagic degradation to transport ferritin into lysosomes.^58^, ^59^ In consideration of the negative feedback of NCOA4 on cytosol iron, overloaded iron, especially polyunsaturated fatty acid‐containing phospholipids, is particularly prone to be peroxidative under exposure to immoderate ferritinophagy.^60^ In response to the accumulated Fe^2+^ induced by upregulated expression of NCOA4 in ferritinophagic response, the consequent downstream signalling cascades are implicated in cytomembrane destruction and even ferroptotic cell death.^61^ Hence, downregulation of NCOA4 in partial or alleviation of ferritinophagy might significantly protect cells from excessive lipid peroxidation, or else unresolved overloaded iron induced by ferritinophagy is related to ferroptosis.

**FIGURE 4:**
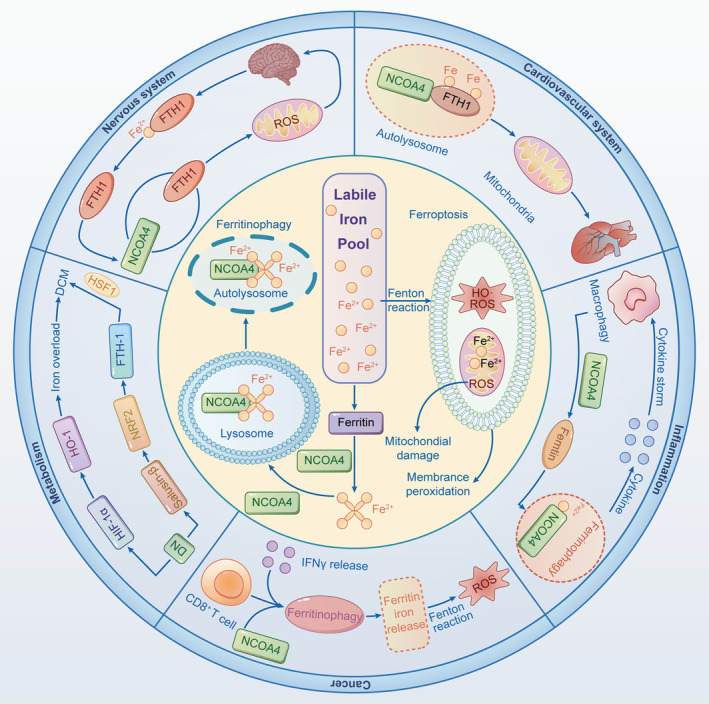
The molecular mechanisms underlying NCOA4‐mediated ferritinophagy in ferritinophagy–ferroptosis axis. NCOA4 refers to ferritinophagy receptor that combined with C terminal of FTH, accounting for the initiation of selective autophagy, special degradation of ferritin and enhanced bioavailability of cytosol iron. NCOA4 can interact with HERC2 to mediate ubiquitination and initiation of autophagic degradation to transport ferritin into lysosomes. As a result of accumulated Fe^2+^ induced by upregulated expression of NCOA4 in ferritinophagic response, the consequent downstream signalling cascades are implicated with cytomembrane destruction and even ferroptotic cell death. In detail, NCOA4‐mediated ferritnophagy and ferritinophagy–ferroptosis axis are involved in human diseases. Elevated level of redox‐active iron owning to transferrin hyperactivation may contribute to abnormal ferritin synthesis in human diseases. FTH1/FTL, ferritin heavy and light chains; HIF‐1α, hypoxia‐inducible factor‐1α; HO‐1, heme oxygenase‐1; HSF1, heat shock factor 1; NCOA4, nuclear receptor coactive 4; NRF2, nuclear factor erythroid2‐related factor 2; ROS, reactive oxygen species.

Another pathway, involved in metabolism‐dependent disorder, is also reported to be responsible for disturbance in ferritinophagic homeostasis. Myo‐inositol oxygenase, as an example, refers to a type of oxidase in renal proximal tubule‐residing, which is documented as an essential contributor to the promotion of ferritinophagy and inactivation of glutathione peroxidase 4, eventually leading to metabolic disorder‐induced ferroptosis.^62^ Indeed, it can be concluded that metabolic imbalance may proceed to aggravate ferroptotic cell death ahead of ferritinophagy disturbance. Also, ferritin protein is capable of producing a direct impact on Fe^2+^‐containing metabolic enzymes by releasing iron to the cytosol.^63^ As a more detailed overview underlying the adverse effect of overactivated ferritinophagy on metabolic disorders and even ferroptosis needs to be further interrogated, we subsequently summarise the possible signalling cascades of ferritinophagy–ferroptosis axis and molecular targets for regulating ferritinophagy as a potential therapeutic in the remaining part (Figure 4).

### 3.2 The signalling pathway and molecular target for ferritinophagy–ferroptosis axis

As mentioned above, inhibition of NCOA4 ferritinophagy can suppress cells from ferroptosis induction under metabolic disorder conditions. It has been reported that *Ncoa4* deficiency is mainly responsible for ameliorating erastin‐induced ferroptosis, whereas enhanced expression of NCOA4 aggravates ferroptotic cell death triggered by RAS‐selective lethal (RSL3).^64^, ^65^ Furthermore, evidence on the crosstalk between ferritinophagy and ferroptosis indicates that overexpression of NCOA4 leads to an increased cytosolic iron upon septic insults, which subsequently renders the upregulation of mitochondrial protein sideroflexin 1 (SFXN1), ultimately presenting with SFXN1‐mediated Fe^2+^ accumulation in mitochondria.^66^ In agreement with this finding, administration of apelin‐13 on the cardiomyocyte hypertrophy model exerts a beneficial effect on ferritinophagic remission, suggesting that knockdown of ferritinophagy‐specific genes, especially NCOA4, may present with extensive aggregation and consequently drive a deterioration of ferroptosis.^67^ Similar to SFXN1, MAPK8, a member of the mitogen‐activated protein kinase (MAPK) family that is liable to be activated under constantly hypoxia exposure, can mediate the structural domain of miR6862‐5P to facilitate NCOA4 mRNA degradation, demonstrating that MAPK8‐MIR6862 is not only involved in the inhibition of NCOA4‐mediated ferritinophagy, but also enhances the expression of mitochondrial ferritin to protect against ferroptosis.^68^ A study of HERC2‐mediated ubiquitination revealed that the ubiquitination of HERC2 enhanced the autophagic degradation of NCOA4, thereby interrupting ferritinophagy and, consequently, ferroptosis.^69^

In addition to the direct impact of NCOA4 on ferritinophagy–ferroptosis axis, the expression level of intracellular FTH is confirmed as another independent element for this signalling cascade. Taking a HepG2 cell model as an example, decreased levels of FTH were shown to markedly promote ferritinophagy and ferroptosis compared with elevated expression of NCOA4.^70^ Evidence generated in both PC cell and Parkinson disease (PD) models showed that FTH overexpression could yield negative feedback on ferritinophagy–ferroptosis axis via downregulating NCOA4 and microtubule‐associated protein 1 light chain 3 alpha.^71^ Thus, FTH might be regulated by special genes in ferritinophagic‐associated autophagy, which is considered a potential target for inhibiting the ferritinophagy–ferroptosis axis in various diseases.

Due to the interplay between ferritinophagic‐associated autophagy and lysosome per se, suppression of ferritin degradation in lysosome may result in inhibition of ferritinophagic response. In accordance with this viewpoint, deficiency in ferritinophagy, as a result of impaired hydrolases transporting into lysosome once the mice were disposed of with synuclein α (SNCA), contributed to the overloaded ferritin accumulated in the outer retina, eventually driving irreversible degeneration of retina in vivo and in vitro.^72^ Conversely, RAB1A overexpression was confirmed to restore the SNCA‐mediated ferritinophagy from the inhibitory response, which demonstrated the capacity of both SNCA and RAB1 in attenuating ferritin degradation and ferroptosis.^72^ As SNCA is involved in the process of non‐selective autophagy to impact protein degradation, it has failed to be recognised as a special receptor to inhibit ferritinophagy.^73^, ^74^ Therefore, targeting ferritinophagy to prevent the targeted effect on overloaded autophagosomes, which is summarised in Table 1, seems to be a promising therapeutic strategy for various diseases.

**TABLE 1:** The regulators of NCOA4‐mediated ferritinophagy in ferritinophagy–ferroptosis axis.

Molecules	Regulatory effects
HERC2	Combining with NCOA4 and promotes proteasome to degrade NCOA4.
FTH1	Facilitating the dissociation of NCOA4–FTH1 complex in iron‐replete condition.
PCBP1	Binding with NCOA4 and ferritin to balance iron homeostasis.
MIOX	Augmenting ferritinophagy and inactivates glutathione peroxidase 4.
SFXN1	Combining with NCOA4 and presents with Fe^2+^ accumulation in mitochondria.
MAPK8	Mediating the structural domain of MIR6862 to facilitate NCOA4 degradation.
SNCA	Contributing to the overload ferritin accumulated in the outer retina.

Abbreviations: FTH1, ferritin heavy chain; HERC2, HECT and RLD domain containing E3 ubiquitin protein ligase 2; MAPK8, mitogen‐activated protein kinase 8; MIOX, myo‐inositol oxygenase; NCOA4, nuclear receptor co‐activator 4; PCBP1, ploy rC‐binding protein 1; SFXN1, mitochondrial protein sideroflexin1; SNCA, synuclein α.

## 4 POTENTIAL LINK OF NCOA4‐MEDIATED FERRITINOPHAGY TO HUMAN DISEASES

Increasing evidence substantiates the effect of NCOA4‐mediated ferritnophagy on human diseases, such as neurodegenerative disorders, cardiac hypertrophy, inflammatory‐related diseases and cancer. Theoretically, we speculate both NCOA4‐mediated ferritnophagy and ferritinophagy–ferroptosis axis are potential therapeutic targets in the treatment of human diseases. NCOA4‐mediated ferritnophagy involved in various human diseases is listed in Table 2.

**TABLE 2:** Ferroptotic tissues and organs presenting necroinflammation.

Systems	Types of diseases	Evidences	References
Nervous system diseases	Alzheimer's disease (AD)	Overloaded iron hindered IRE from binding APP region and enhanced β‐amyloid production.	75, 76, 77, 78
	Parkinson's disease (PD)	Transferrin hyperactivation and divalent metal transporter 1 (DMT1) overexpression contributed to abnormal ferritin synthesis.	79, 80, 81, 82
	Huntington's disease (HD)	Downregulated NCOA4 exerted a protective impact on neurocytes in the setting of HD.	75, 83, 84
Cardiovascular system diseases	Heart failure (HF)	Driven cardiomyocyte remodelling to take cardiomyocyte into a transverse aortic constriction condition.	85
	Hypertrophic cardiomyopathy	Promoted ferritinophagy‐related free iron and mitochondrial iron depletion.	4, 59
	Ischaemia/reperfusion injury	Oxygen–glucose deprivation improved baicalin administration.	86
Inflammatory diseases	Urinary tract infections (UTIs)	*Escherichia coli* shuttled into the autophagosome and lysosome to combine with iron.	63
	Periodontal ligament fibroblasts (PDLFs)	Butyrate depended on HIF‐1α pathway activation and CDK9 coordination to trigger NCOA4‐mediated ferritinophagy.	59, 87
	Sepsis‐induced cardiac injury	LPS stimulation upregulated NCOA4 expression to combine with ferritin and increase Fe^2+^ in mitochondria.	88
	Coronavirus disease 2019 (COVID‐19)	High levels of cytokine storms resulted in hyperferritinemia and amplified inflammation.	89, 90, 91, 92, 93
Metabolism‐related diseases	Diabetes	High glucose induced the upstream core regulatory substances into a mutability, and resulted in ferroptosis.	94, 95, 96
	Glucocorticoid‐induced osteoporosis	Endothelia cell‐secreted exosomes suppressed ferritinophagy–ferroptosis axis and reversed glucocorticoid‐induced osteogenic inhibition.	97
Cancer	Clear cell renal cell carcinoma (ccRCC)	Elevated NCOA4 expression and ferritin degradation in glioblastoma.	98, 99, 100
	Melanoma	Lack of BNIP3 in melanoma cells induced increases in intracellular iron levels.	101, 102
	Glioblastoma	Ferroptotic inhibitors (erastin and RSL3) triggered ferroptosis in neuroblastoma N2A cells rather than normal nerve cells.	103, 104

### 4.1 Nervous system disease

A growing number of evidence has implicated NCOA4‐mediated ferritinophagy to be a major cause of the pathogenesis of neurodegenerative disorders, including Alzheimer's disease (AD), PD, Huntington's disease and amyotrophic lateral sclerosis. As we all know, iron is regarded as an indispensable element for physiological function in the brain, including oxygen delivery, mitochondrial energy supply as well as neurotransmitter secretion.^75^ Accordingly, many studies have consistently put forward an in‐depth review that both dysfunctions of iron homeostasis and protein quality control are the critical mechanisms underlying neurodegenerative disorders, which present similar importance towards the ferritinophagy–ferroptosis axis. As far as neurodegenerative diseases are concerned, deficiency of ferritinophagy attributed to genetic perturbation in lysosomes may result in a high susceptibility to ageing of brain tissue.^76^, ^77^ As reported in 2018, WIPI4 protein, referring to WDR45 encodes WD‐repeat domain 45 protein, was confirmed to play a vital role in the initiative progression of autophagy, as evidenced by autophagic deficiency, lysosomal dysfunction, iron overload and oxidative injury under the exposure to pathogenic mutations in WDR45.^78^ In support of this viewpoint, Saitsu et al. noted multiple iron and peroxidase deposited in globus pallidus and substantia nigra while suffering from WDR45 dysfunction, which could be recovered by the pharmacologic administration of autophagy pathway.^105^ Given that NCOA4‐mediated ferritinophagy has been noticed to implicate in iron homeostasis and classical autophagic pathway, it is hypothesised that ferritinophagy deficiency might be the underlined mechanism for improper iron deposition in neurodegenerative diseases.

AD is considered one of the most common causes of cognition impairment in neurodegenerative diseases, and it is characterised by high concentrations of reduced‐state compounds, such as iron, deposited in aggregates to result in neuronal dysfunction.^79^, ^106^ On account of excessive amyloid precursor protein (APP) that owns a presumptive iron response element (IRE) in its untranslated region to regulate the post‐transcriptional system, overloaded iron might hinder IRE from binding with APP region and subsequently enhance APP translation to facilitate β‐amyloid (Aβ) production at last.^80^ In fact, inappropriate accumulation of redox‐active metallic elements, especially iron, is regarded as the critical pre‐requisite for the accelerated progression of AD, which has reportedly been considered a therapeutic target for neurodegenerative diseases in clinical trials.^81^ This notion has been evidenced by the current phase II clinical trials, indicating that chelation therapy for patients with AD can effectively decrease Aβ deposition in plasma and cerebrospinal fluid to further improve prognosis.^82^, ^83^ Another common neurodegenerative disease secondary to AD, PD is featured on aggregated SNCA, mitochondrial disorders, oxidative impairment and high concentration of iron in substantia nigra.^75^, ^84^ Mechanically, elevated levels of redox‐active iron owning to both Tf hyperactivation and divalent metal transporter 1 overexpression contribute to abnormal ferritin synthesis in PD patients, ultimately leading to a poor outcome.^13^, ^107^ Given that redox‐active iron is closely related to the severe neuronal injury resulting from the overgeneration of oxidase, improper iron stockpile might potentially be the leading link to the pathogenesis of PD.

Recently, the central function of NCOA4‐mediated ferritinophagy in neurodegenerative diseases has attracted more attention, as evidenced by the high expression of NCOA4 in murine brain tissue at both mRNA and protein levels.^19^ It is presumed that ferritinophagy flux deficiency is deemed to be observed as it is accompanied by a decreased level in globe autophagy. In accordance with *Ncoa4*‐KO model in vitro, a depletion in bioavailable iron was found on account of decreased ferritin degradation. Consequently, it resulted in attenuated oxidative stress and lower sensitivity to ferroptotic cell death, indicating that downregulated NCOA4 could exert a protective impact on neurocytes in the setting of neurodegenerative diseases.^108^ Nonetheless, the discrepancy in research findings between in vitro and in vivo remains noteworthy, resulting from different extents of *Ncoa4* deficiency, disparate growth conditions of cells and various independent degrees of brain tissue on NCOA4‐mediated ferritinophagy. In a murine model of liver injury with *Ncoa4*‐KO, for example, accumulated iron together with high sensitivity to oxidative stress was remarkably observed in not only live tissue but also in splenic organs.^85^ As demonstrated above, it is speculated that continuous depletion of NCOA4 in brain tissue in ND animal models may deteriorate phenotype attributing to inappropriate deposition, in turn leading to oxidative‐associated stress, including ferroptosis. Taken together, more convincing evidence underlying the expression level of NCOA4 in the brain and the regulated mechanism in neurocytes are required to further illustrate the targeting of function of NCOA4 for neurodegenerative disorders.

### 4.2 Cardiovascular system disease

It is accepted that dysregulated iron homeostasis has been implicated in various cardiovascular diseases for the reason that excessive iron may augment the release of inflammatory mediators, which jeopardise cardiomyocytes and even interdict mitochondrial energy supply.^4^, ^86^ Recently, evolving studies have documented that NCOA4‐mediated ferritinophagy plays a central role in ferroptosis initiation and drives significant pathological processes in cardiovascular diseases, such as heart failure (HF), cardiac hypertrophy and cardiac ischaemia/reperfusion (I/R) injury.^63^, ^87^ First, NCOA4‐mediated ferritinophagy is reportedly capable of driving the cardiomyocyte remodelling to further take cardiomyocyte into a transverse aortic constriction (TAC) condition, which is characterised by suppression of left ventricular diastolic function and myocardial fibrosis.^59^ Accordingly, special markers for cardiac remodelling exhibit remarked upregulation once the cardiomyocytes are conducted with TAC induced by NCOA4 overexpression. As soon as TAC was reversed in NCOA4 deficient mice, FTH levels in cardiomyocytes showed a significant decrease and subsequently hypoactivation in ferritinophagy.^59^ Therefore, NCOA4‐mediated ferritinophagy is deemed to have great potential in pressure overload‐induced cardiac remodelling. Second, the apelin/APJ system, regarded as a cardiac pro‐hypertrophic stimulus, is generally taken advantage of by facilitating hypertrophy of cardiomyocytes, owing to its ability to promote ferritinophagy‐related free iron and mitochondrial iron depletion.^88^ Of note, as described in the previous section, apelin‐13 can transfer iron to mitochondria with the help of SFXN1, leading to ferritinophagy‐induced iron overload and consequently triggering myocardial hypertrophy.^64^ Third, a recent article by Fan et al. reported that both exasperated ferroptosis and hyperactive ferritinophagy emerged in the myocardial I/R model together with oxygen–glucose deprivation, which might be improved by baicalin administration, indicating that NCOA4‐mediated ferritinophagy acts as bridges to connect free iron and myocardial metabolism.^89^ More advances in exploring ferritinophagy‐associated molecular pathways are required for not only HF, cardiac hypertrophy and myocardial I/R injury but also other cardiovascular diseases to further understand the aetiology of cardiovascular diseases and pay more attention to potential ferritinophagy–ferroptosis axis targeting as novel approaches for therapy.

### 4.3 Inflammatory disease

As we know, lysosomal damage due to iron overload is demonstrated as the specific mechanism for causing dysfunction of immune cells and ferroptotic cell death in the setting of inflammatory diseases. In fact, host cell death and bacterial burden can be reversed by inhibition of iron‐regulated selective autophagy. In a previous study, NCOA4‐mediated ferritinophagy was proposed to be involved in the pathophysiology of urinary tract infections (UTIs) induced by uropathogenic *Escherichia coli* (UPEC). It was documented for the first time that UPEC possessed a strong ability in shuttling into the autophagosome and lysosome in the phase of combination with iron. They then confirmed NCOA4‐dependent manner could enhance iron availability for UPEC, driving bacteria overproliferation and ferroptotic cell death, which suggested ferritin dysfunction and overactivated ferritinophagy to be the major cause for recurrent UTIs.^90^ Subsequent study on periodontal ligament fibroblasts revealed a similarity towards the perspective that butyrate (an important short‐chain fatty acid in the periodontal pocket) triggered NCOA4‐mediated ferritinophagy, which depended on p38 MAPK/hypoxia‐inducible factor‐1α pathway activation and cyclin‐dependent kinase 9 coordination.^91^ In complete agreement with this notion, Guo's teamwork found upregulated expressions of NCOA4, ferritin heavy chain and light chain in infective periodontal tissues, which implied that *P. gingivalis* infection resulted in NCOA4 upregulation to enhance LIP and ROS accumulation.^92^

Until 2020, a study by Li et al. elaborately verified that sepsis‐induced cardiac injury presented close contact with the protein level of NCOA4 and ferritin degradation when H9c2 myofibroblasts were stimulated by lipopolysaccharide (LPS), as evidenced by the expressed level of ferritin significantly decreased with a time dependence whereas NCOA4 expression initiated to be increased upon LPS treatment. Moreover, increased cell survival and reduced lipid peroxidation could be obviously observed in H9c2 myofibroblasts when NCOA4 was downregulated.^93^ Interestingly, in addition to elevated levels of ferroptotic marker cyclooxygenase‐2, LPS also upregulated NCOA4 expression to immediately bind up to ferritin, subsequently leading to an increased Fe^2+^ in mitochondrial and overproduction of mitochondrial ROS.

Notably, bacteria‐induced infection appears a close crosstalk with ferritinophagy, virus infection, for example, coronavirus disease 2019, can also trigger hyperferritinemia, which is characterised by severe cytokine storms and aggravated inflammatory response.^109^, ^110^ High levels of the inflammatory state featured in various cytokine storms result from SARS‐CoV‐2 invasiveness. These cytokine storms give rise to hyperferritinemia, thereby immortally magnifying the infective reaction.^111^ Accompanied by the combination of NCOA4 with ferritin and delivery into autophagosomes for degradation, multiple superoxides and even ferroptosis are generated by the excess of intracellular iron, consequently resulting in severe tissue injury.^94^, ^95^ Realistically, autophagic deficiency appears to be more susceptible to infectious diseases in the case of a genetically insufficient model, for the reason that inactive autophagy exactly contributes to the lower level of inflammatory cytokines release.^96^ More interestingly, genetic mouse models have shown to be more sensitive to iron accumulation accompanied by a hyper lever of serum iron, which revealed blocking the process of ferritinophagy can significantly give rise to iron overload even though the unavailability of iron for erythropoiesis. Therefore, these lines of evidence have provided a novel molecular insight into therapeutic strategy from the perspective of interfering with both inflammatory cytokine and ferritin degradation.

### 4.4 Metabolism‐related diseases

Recently, accumulating evidence has suggested that metabolism‐related diseases commonly proceed with a disorder of cell metabolic pathway, ferritinophagy and even ferroptosis.^97^, ^112^, ^113^ Taking diabetes as an example, it is documented that diabetic complications generally occur with the initiation of NCOA4‐mediated ferritinophagy, which further increases the degradation of lysosomal proteins and drives the body to a high susceptibility to insulin resistance.^114^ As a result of high glucose taking the upstream core regulatory substances into a mutability, antioxidant capacity presents with a remarkable inhibition and it ultimately leads to ferroptosis.^115^, ^116^ At the initial stage in the diabetic myocardial model, it showed a sign of hyperactivated ferritinophagy and increased release of iron, which was different from classical autophagy. Subsequently, enhanced expression of NCOA4 along with a higher level of DNA (cytosine‐5)‐methyltransferase 1 (DNMT‐1) that referred to an epigenetic modification‐associated enzyme was obviously detected, hinting that blockade of DNMT‐1 not only relieved the left ventricular dysfunction but also regulated ferritinophagy–ferroptosis axis in myocardial tissue.^117^

A recent report on the model of glucocorticoid‐induced osteoporosis appeared to be a noteworthy issue with regard to exosomes derived from vascular endothelial cells. It demonstrated endothelial cell‐secreted exosome, an important mediator of cell‐to‐cell communication, to suppress the ferritinophagy–ferroptosis axis and further reverse glucocorticoid‐induced osteogenic inhibition.^118^ In the future, systematical comprehension of ferritinophagy–ferroptosis axis focusing on their potential mechanisms, connection and regulation in metabolism‐related diseases seems to be necessary and will lay the foundation for emerging therapeutic opportunities as well as associated challenges with long‐term prospects.

### 4.5 Cancer

In recent years, it has become increasingly clear that iron metabolism disorders in various types of tumours and functions and properties of iron in ferroptosis, as well as ferritinophagy, are developing into the latest hotspot in the field of cancer research. As illustrated in previous studies, ferritinophagy‐related genes have been confirmed as the indispensable pre‐requisite for both cancer progression and tumour cell proliferation^98^, ^99^, ^100^, ^101^, ^102^, ^103^, ^119^; here, we mainly focus on clarifying the new discoveries on the interplay between NCOA4 and FTH in various types of cancer. In the model of clear cell renal cell carcinoma, it was noted a decreased expression of NCOA4 had an impact on high‐grade malignant tumours and advanced TNM staging.^104^, ^120^ Given that NCOA4‐mediated ferritinophagy plays a key role in tumour proliferation and differentiation, researchers have demonstrated COPZ1 downregulation to elevate NCOA4 expression, in turn contributing to ferritin degradation and ferroptosis in glioblastoma.^121^ Besides, some studies have documented that a lack of BNIP3 in melanoma cells can lead to increased intracellular iron levels, ROS depletion and peroxidative stress attributed to hyperactivation of NOCA4‐mediated ferritinophagy.^122^ Intriguingly, Hasan et al. found that not all cancer cells presented a reactivity to NOCA4‐mediated ferritinophagy, such as colon cancer cells, which revealed a discrepancy between normal circumstances and iron‐limited surrounding as soon as NCOA4 KO, indicating that NCOA4 failed to participate in erastin‐induced ferroptosis.^123^

Another condition precedent to impact the progression of NOCA4‐mediated ferritinophagy is reportedly to be FTH. For example, cystine deprivation resulted in ferroptosis in glioblastoma cells, as Hayashima et al. found that NCOA4‐mediated ferritin degradation and Tf delivery into lysosome were deemed to be remarkably observed in cystine‐deprived cells.^124^ In addition, ferritinophagy has been verified to play a critical role in cystine deprivation‐mediated ferroptosis for the reason that interference with FTH degradation can suppress tumour cells from cystine deprivation‐induced ferroptosis.^124^ Notably, ferroptotic inhibitors, including erastin and RSL3, trigger ferroptosis in neuroblastoma N2A cells rather than in normal nerve cells, implicating that ferroptosis is bound to be a promising therapeutic target for neuroblastoma.^125^ From these perspectives, signalling modulation of the NCOA4‐mediator ferritinophagy–ferroptosis axis may generate a hopeful strategy for drug‐resistant cancer cells on account of different sensitivity to ferritinophagy and response to ferroptosis.

## 5 CONCLUSIONS AND PERSPECTIVES

NCOA4‐mediated ferritinophagy indubitably reveals various benefits in defending against human diseases through iron transport and ferritin degradation, thereby improving the prognosis and survival of cells. Nevertheless, excessive ferritinophagy may inevitably cause overload iron, accumulated peroxidations and further result in disruption of cellular function and viability to some extent. Although the latest version has recently highlighted the vital role of ferritinophagy in human diseases, in‐depth studies are warranted to elucidate the molecular interaction and regulated machinery of ferritinophagy. First, the outstanding challenge regarding the biochemical regulation of NCOA4 and the manner in which NCOA4 is regulated on the transcriptional level has yet to be explored. From molecular perspectives, in spite of the NCOA4‐FTH cascade response being impacted by intracellular iron level, the pattern of NCOA4 binding with iron as well as the structural domain, however, remains undefined. Indeed, FTH overload and even aggregation might be infinitely aggravated followed by excessive accumulation of endogenous iron as well as FTH dysfunction. Furthermore, the inter‐coordination between subcellular organelles, lysosomes and autophagosomes is regarded as the key link in maintaining ferritinophagy homeostasis, which provides a more detailed explanation for dyshomeostasis observed in multiple diseases. Second, because NCOA4 acts as a special regulator located in the nucleus involved in nuclear receptor co‐activation and DNA replicative initiation, the precise canonical nuclear location sequence is required in the following study. Third, further exploration of the effect of NCOA‐mediated ferritinophagy on multiple organs and immune systems in the setting of NCOA4 conditional KO appear to be more critical. Given that NCOA4 has been demonstrated to act as a regulated function in genome stability, protein structure prediction and cell differentiation, it is reasonable to believe in the importance of NCOA4‐mediated ferritinophagy for iron‐dependent cellular processes. With respect to clinical application, we are looking forward to investigating an appropriate construction to improve host responses by modulating the sensitivity to ferritinophagy. In the future, the maintenance of cellular iron homeostasis through a ferritinophagy‐related pathway is considered a potential therapeutic strategy for multiple diseases because hyperflux iron might result in more sensitivity to ferroptosis‐induced agents. It is our belief that a deep understanding of the ferritinophagy–ferroptosis axis might promote a surge in research on the quality control of multiple organs and arouse broad concerns about intracellular self‐protective instinct. Therefore, it is reasonable to shed light on synchronously modulating the activity of NCOA4‐mediated ferritinophagy and its underlying pathway, which might be of great significance in developing novel interventional strategies for various human diseases.

## AUTHOR CONTRIBUTIONS

Y‐MY, R‐QY and Y‐PT conceptualised, supervised and revised the manuscript. J‐YL and Y‐HF conducted the literature review and drafted the paper. Y‐XL, P‐YH and Q‐YZ helped with preparing the manuscript. All authors read and approved the final manuscript.

## CONFLICT OF INTEREST STATEMENT

The authors declare no conflict of interest.

## Data Availability

Further information and requests for resources and regents should be directed to and will be fulfilled by the Lead Contact, Yongming Yao (c_ff@sina.com).
